# BRAF V600E and lymph node metastases in papillary thyroid cancer

**DOI:** 10.1530/EC-20-0420

**Published:** 2020-09-16

**Authors:** Pan Chen, Liqin Pan, Wensi Huang, Huijuan Feng, Wei Ouyang, Juqing Wu, Jing Wang, Yuying Deng, Jiaxin Luo, Yanying Chen

**Affiliations:** 1 Department of Nuclear Medicine, Zhujiang Hospital of Southern Medical University, Guangzhou, Guangdong Province, China

**Keywords:** BRAF V600E, lymph node, thyroid carcinomas, pathological characteristics

## Abstract

**Objective:**

To evaluate the relationship between the BRAF V600E mutation in lymph node metastasis (LNM) and its invasive characteristics in papillary thyroid cancer (PTC).

**Material and methods:**

A total of 373 PTC patients were enrolled in this study conducted at Zhujiang Hospital of Southern Medical University between January 2017 and December 2018. PTCs with cervical lymph node metastases were verified pathohistologically, and primary tumors and LNM were examined for the BRAF V600E mutation. Patients were excluded from the study if the BRAF V600E mutation was examined only in primary tumors or only in LNM.

**Results:**

Of the 373 patients examined, BRAF V600E mutation frequency in primary tumors was slightly higher than in LNM (81.5% vs 78.0%, *P* = 0.000), the intra-class correlation coefficient (ICC) was 0.865 (95% CI 0.835–0.890). The BRAF V600E mutation in both primary tumor and LNM negatively correlated with the size of the largest metastatic focus of LNM (Odds ratio, OR = 0.297, 95% CI 0.143–0.616, *P* = 0.001; OR = 0.242, 95% CI 0.119–0.492, *P* = 0.000, respectively). There was no relationship between BRAF V600E mutation in LNM and the number, extranodal extension or stage of LNM (*P* > 0.05).

**Conclusion:**

The BRAF V600E mutation in LNM may not be related to the invasive characteristics of LNM in PTC.

## Introduction

Over the last decades, thyroid cancer has been the fastest growing and most prevalent endocrine malignancy worldwide. The Cancer Center of China has recently reported that thyroid cancer was the eighth most common cancer and fourth most common malignant tumor among women (1). More than 95% of thyroid cancer cases involve differentiated thyroid cancer (DTC) and up to 90% belong to papillary thyroid cancer (PTC) (2).

The BRAF V600E mutation is the most common genetic alteration in PTC, resulting in abnormal cell proliferation and carcinogenesis via the MAPK/ERK pathway (3). Multiple studies have found that the BRAF V600E mutation in primary tumors is associated with pathological invasiveness, recurrence, and mortality (4, 5, 6). Moreover, the BRAF V600E mutation has been incorporated into the recurrence risk stratification of the 2015 American Thyroid Association (ATA) guidelines (7).

Despite our current knowledge of the role of BRAF V600E in primary tumor in tumorigenesis of PTC, considering tumor heterogeneity, the relationship between BRAF V600E and clinical characteristics should also be evaluated in metastases. Moreover, some scientists hope that we may extend our observation to metastatic PTC to generate a critical mass of data for clinical practice (8). However, only a few studies have examined BRAF V600E mutation in metastases, such as lymph node metastasis (LNM) (9, 10). The PTC prognosis is usually favorable with appropriate treatment, however, LNM can develop during early stages of the disease (11). Cervical lymph node metastasis is common in PTC, with approximately 20–90% of patients having LNM at initial presentation (12, 13). The presence of clinically evident neck LNM has been shown to be a predictor of persistent or recurrent disease during follow-up (14). Prognosis depends on pathological characteristics of LNM, such as size, number, and extranodal extensions (15, 16). In the era of personalized medicine and systematic targeted treatment of diseases, it is essential to examine molecular changes in metastases to accurately assess prognosis. Therefore, further information on the BRAF V600E mutational status of LNM and its pathological features is highly required. The purpose of the current study was to determine the nature of the relationship between the BRAF V600E mutation in LNM and the pathological features of LNM.

## Materials and methods

### Patients

**Figure 1:**
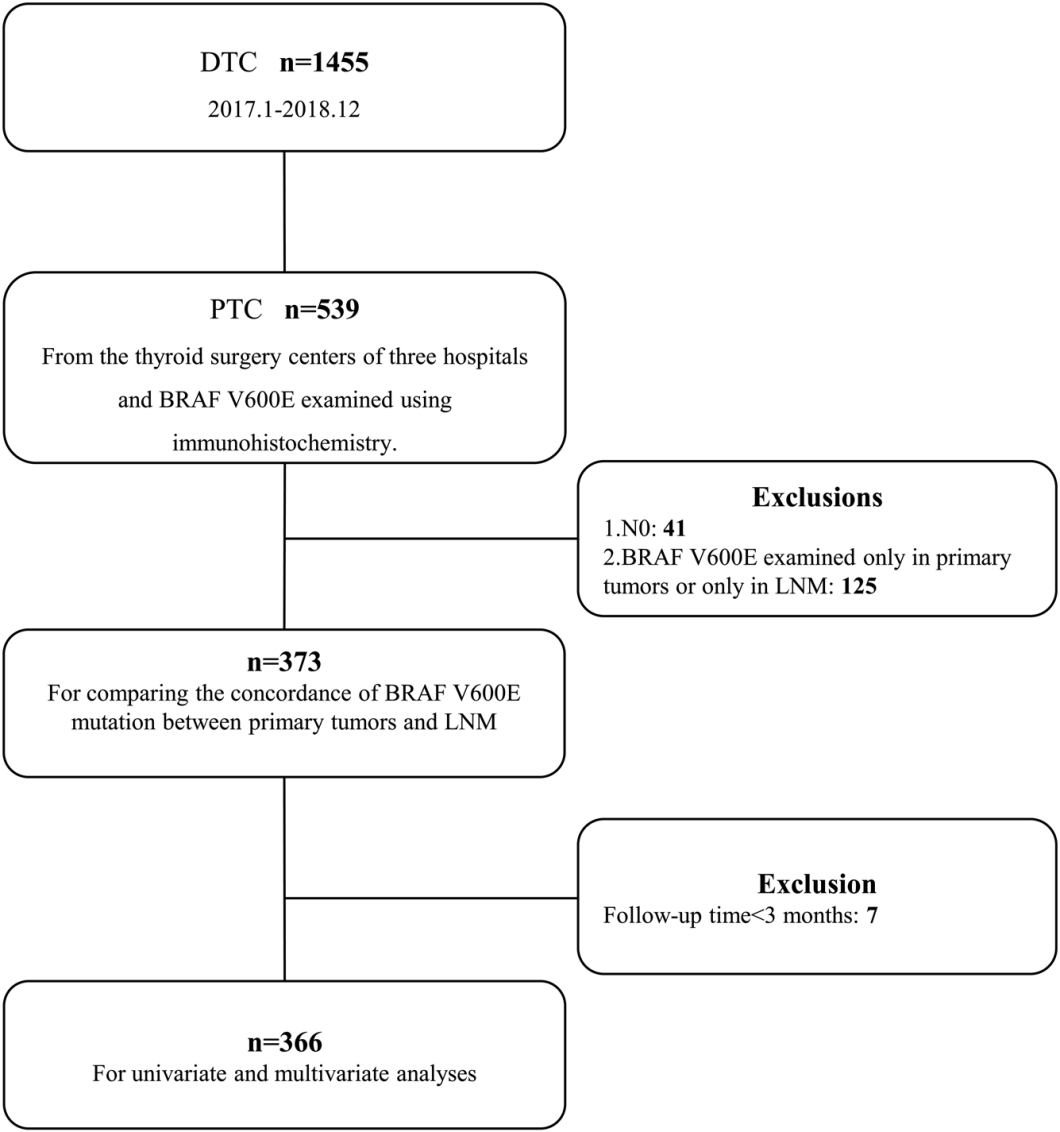
Flowchart indicating study inclusion and exclusion criteria. DTC, Differentiated thyroid cancer; PTC, papillary thyroid cancer; LNM, lymph node metastasis; N0, pathologically proven no cervical lymph node metastasis.

Our study was approved by the Ethics Committee of Zhujiang Hospital of Southern Medical University. We obtained informed consent from each patient after a full explanation of the purpose and nature of all study procedures. Our department is the center of radioactive iodine (^131^I) treatment of thyroid cancer in South China. Postoperative DTC patients from more than ten other hospitals came to our department for further diagnosis and treatment. Among a total of 1455 DTC patients who visited our department between January 2017 and December 2018, more than one-third (*n* = 539) PTC patients came from the thyroid surgery centers of three hospitals. Patients in these three hospitals had the BRAF V600E gene status examined using a consistent detection method (immunohistochemistry). Therefore, we selected these 539 patients from the three hospitals. Patients were included if they fulfilled the following criteria (Fig. 1): (i) PTC with cervical lymph node metastasis was proven pathohistologically; (ii) BRAF V600E was detected both in primary tumor and LNM; and (iii) Follow-up was done for a minimum of 3 months. Patients were excluded from the study if they BRAF V600E mutation status was only examined in primary tumors or only in LNM. Finally, a total of 373 patients were enrolled in this study and 366 were enrolled when analyzing therapeutic response.

We collected the patients’ clinical data, including general characteristics (diagnosis age, gender and family history), pathological characteristics of primary tumors (histological variant, tumor size, multifocality, lesion location, extrathyroidal extension, capsular invasion), pathological characteristics of LNM (stage, number, size of the largest metastatic focus of LNM, extranodal extension), distant metastases, the BRAF V600E status in primary tumor and in LNM, the ^131^I cumulative dose, risk stratification and therapy response according to 2015 ATA guidelines (7). N stage according to the eighth edition of the American Joint Committee on Cancer (AJCC) Tumor Node Metastasis (TNM) staging system and size of the largest metastatic focus of LNM refered to the largest dimension of the focus filled with metastatic thyroid cancer in LNM observed microscopically in multiple serial sections. Capsular invasion referred to the invasion of the thyroid capsule, but limited to the thyroid gland, whereas extrathyroidal extension referred to extensions beyond the thyroid capsule to invade the surrounding soft tissues.

### Treatment protocol

All of the patients underwent thyroidectomy, neck LN dissection and TSH suppression according to 2015 ATA guidelines (7). The patients followed a low-iodine diet for 3–4 weeks and thyroid hormone withdrawal at least 3 weeks before ^131^I therapy. The oral ^131^I dose depended on the postoperative risk stratification and stimulated thyroglobulin (sTg) level of each patient. A total of 299 patients underwent either thyroid remnant ablation or adjuvant therapy, and 11 patients underwent two or more sessions of ^131^I therapy due to persistent radioiodine-avid lesions or elevated sTg (generally >10 ng/mL) with negative radioiodine imaging and no 18F-fluorodeoxyglucose (18F-FDG) uptake. Following the ^131^I therapy, ^131^I whole-body scan was performed after 2–7 days, and TSH suppression was implemented for all patients.

### Follow-up protocol and therapy response system

Patient follow-up was done every 3–6 months during the first 2 years after surgery, and then every 6–12 months when the condition stabilized. The routine follow-up protocol consisted of serum Tg, anti-thyroglobulin antibody (TgAb), and TSH measurement, as well as the neck ultrasound. A diagnostic whole-body scan (Dx-WBS) was done in patients with incomplete response 6–12 months after the first ^131^I therapy. A 18F-fluorodeoxyglucose PET/CT (18F-FDG PET/CT) scan was performed if the radioiodine imaging was negative, and either serum Tg was elevated or TgAb value was rising.

In total, 366 patients were classified into the following categories (7): complete response, structural incomplete response, biochemical incomplete response and indeterminate response. Complete response was defined as negative follow-up imaging and either suppressed Tg <0.2 ng/mL or sTg <1 ng/mL. Structural incomplete response was defined as structural or functional evidence of disease with any Tg and TgAb level. Biochemical incomplete response was defined as negative imaging and suppressed Tg ≥1 ng/mL or sTg ≥10 ng/mL or rising TgAb levels. Indeterminate response was defined as nonspecific findings on imaging studies, such as a faint uptake in thyroid bed on follow-up radioiodine scan, suppressed Tg between 0.2 and 1 ng/mL, sTg between 1 and 10 ng/mL, stable or declining TgAb in the absence of structural or functional disease.

### BRAF V600E detection

**Figure 2:**
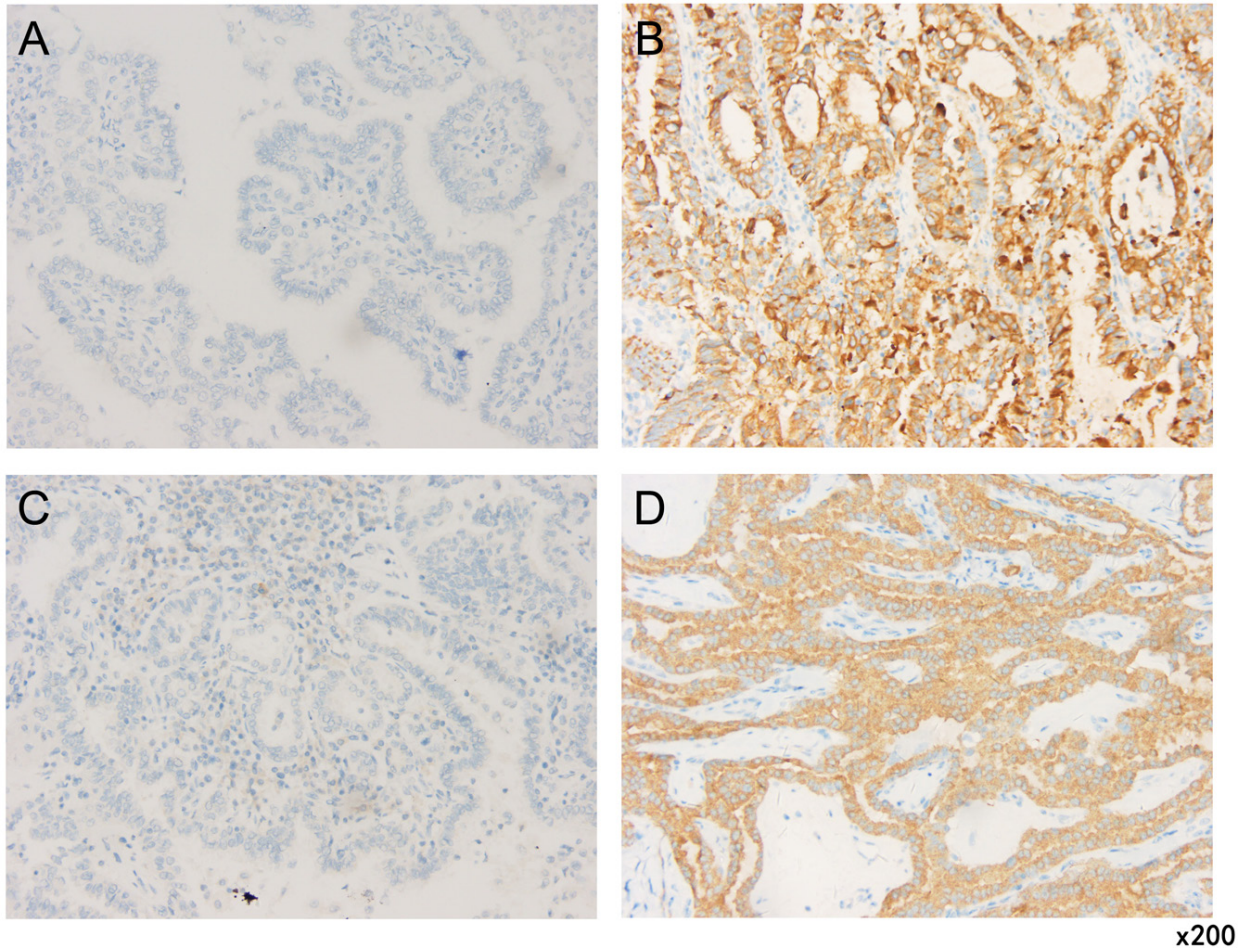
BRAF V600E immunohistochemistry in PTC (×200). (A and C) Non-specific staining in tumor cells (A) and LNM (C), BRAF V600E expression is negative. (B and D) Cytoplasmic staining in tumor cells (B) and LNM (D), BRAF V600E expression is positive. PTC, papillary thyroid cancer; LNM, lymph node metastasis.

Tissue samples from patients with PTC were obtained from postoperative pathological tissues. At least one tissue sample from the primary lesion and one from LNM were obtained for BRAF V600E assessment from each patient. Immunohistochemistry analysis was performed to detect the BRAF V600E mutation, using mouse anti-BRAF V600E (clone VE1, 1:4 dilution, Ventana Medical Systems, Tucson, AZ, USA). The staining procedure was performed using a Benchmark ULTRA autoimmunostainer (Ventana) in accordance with the manufacturer’s instructions. The cells were conditioned for 64 min after which the antibody was added, and the cells incubated at 36°C for 16 min. The cells were then counterstained with hematoxylin-II for 4 min and stained blue for 4 min. Slides were observed under a microscope (BX43, Olympus). For negative control, we omitted the primary antibody, while the positive control was PTC tissue. Nonspecific staining of the colloid and ambiguous weak or focal cytoplasmic staining were considered negative (Fig. 2A and C) while diffuse homogeneous cytoplasmic staining in tumor cells was considered positive (Fig. 2B and D).

### Statistical analysis

Data were analyzed using SPSS 21.0 software (IBM). Measurement data were presented as mean and s.d. or median and range. Categorical variables were compared between two groups using Pearson χ^2^ test. The status of BRAF V600E mutation was compared between primary tumors and LNM using the paired χ^2^ test (McNemar test) and the intra-class correlation coefficient (ICC) consistency test. Univariate and multivariate logistic regression analyses were used to evaluate the relationship between clinical characteristics and the BRAF V600E; the odds ratio (OR) value and 95% CI were reported. *P*-value < 0.05 was considered to be statistically significant.

## Results

### Description of cohort characteristics

Demographic data of all patients included in the study are listed in Table 1. The median age at diagnosis was 37.0 years. The female/male ratio was 1.80:1. A total of 310 patients underwent one or more sessions of ^131^I therapy, 63 patients did not undergo ^131^I therapy, and the median cumulative iodine dose was 4810 MBq (130 mCi). Patients were followed up for 3–39 months (average 24.12 months).

**Table 1:** Baseline characteristics of all PTC patients.

Characteristics	No. (%)
Age at diagnosis (range, years)	37 (16–74)
Sex	
Male	133 (35.7)
Female	240 (64.3)
Family history	
Yes	15 (4.0)
Histologic variants (PTC)	
Classic	361 (96.8)
Follicular	5 (1.3)
Other variants^a^	7 (1.9)
Tumor size (range, cm)^b^	1.2 (0.08–6.0)
Multifocality	188 (50.4)
Lesion location	
Unilateral	190 (50.9)
Bilaterality	183 (49.1)
Capsular invasion	202 (54.2)
Extrathyroidal extension	
No	248 (66.5)
Minimal	98 (26.3)
Gross	27 (7.2)
Distant metastasis	11 (2.9)
N stage^c^	
N1a	190 (50.9)
N1b	183 (49.1)
Number of LNM (range)	5 (1–47)
Size of the largest metastatic focus to the LN (range,cm)^d^	0.4 (0.03–4.0)
Extranodal extension	127 (34.0)
BRAF V600E mutation (primary tumors)	304 (81.5)
BRAF V600E mutation (LNM)	291 (78.0)
^131^I therapy	
No	63 (16.9)
Yes	310 (83.1)
Cumulative iodine dose (range, MBq)	4810 (1295–14,837)
Risk stratification	
Low	41 (11.0)
Intermediate	288 (77.2)
High	44 (11.8)
Response to therapy	
Excellent response	187 (51.1)
Indeterminate response	75 (20.5)
Biochemical incomplete response	28 (7.7)
Structural incomplete response	76 (20.8)
Follow-up time (month)	
Mean (s.d.)	24.12 ± 9.96
Median (range)	25.0 (3–39)

^a^Other aggressive variants include oxyphilic, diffuse sclerosing and solid variant. ^b^Tumor size is recorded as the greatest tumor dimension. ^c^N1a = Metastases to Level VI or VII, N1b = Metastases to Level I, II, III, IV, or V. ^d^Size of the largest metastatic focus to the LN is defined as a focus filled with metastatic thyroid cancer in LNM.

LNM, lymph node metastasis; PTC, papillary thyroid cancer.

### Concordance of BRAF V600E status between the primary tumor and LNM

The BRAF V600E mutation in primary tumors was slightly higher expressed than that in LNM (81.5 vs 78.0%, respectively). Furthermore, there was a significant association between the presence of the BRAF V600E mutation in primary tumors and LNM (*P* = 0.000), and ICC was 0.865(95% CI 0.835–0.890). The concordance of BRAF V600E between primary tumors and LNM is summarized in Table 2.

**Table 2:** Concordance of BRAF V600E between primary tumors and LNM.

	BRAF V600E in primary tumors		*P*	ICC	95% CI
	WT	Mutation			
BRAF V600E in LNM					
WT	61	21	0.000	0.865	0.835–0.890
Mutation	8	283			

PTC, papillary thyroid cancer; LNM, lymph node metastasis; ICC, intra-class correlation coefficient; WT, wild type.

### Univariate and multivariate logistic regression analyses

The results of univariate analysis are shown in Table 3. Patients with larger size and multifocality showed higher BRAF V600E mutation frequency in primary tumors, patients with bilateral lesions and extrathyroidal extension showed higher BRAF V600E mutation frequency in LNM. In PTC patients with other variants, distant metastases, fewer number, and larger size of the largest metastatic focus of LNM, the BRAF V600E mutation frequency was lower in both primary tumors and LNM (all *P* < 0.05). Further multivariate logistic regression analyses (Table 4) found a negative correlation between the BRAF V600E status mutation in LNM and aggressive variants (OR = 0.319, 95% CI 0.113–0.895, *P* = 0.030). The BRAF V600E mutation in primary tumors and LNM significantly negatively correlated with presence of distant metastases (OR = 0.201, 95% CI 0.041–0.993, *P* = 0.049; OR = 0.125, 95% CI 0.023–0.668, *P* = 0.015, respectively) and size of the largest metastatic focus of LNM (OR = 0.297, 95% CI 0.143–0.616, *P* = 0.001; OR = 0.242, 95% CI 0.119–0.492, *P* = 0.000, respectively). There was no relationship between the presence of the BRAF V600E mutation in LNM and number, extranodal extension, stage of LNM or clinical outcomes (*P* > 0.05).

**Table 3:** Univariate analysis of the factors associated with BRAF V600E in primary tumors and LNM.

Characteristics	BRAF V600E in primary tumors			BRAF V600E in LNM		
	WT (*n* = 66)	Mutation (*n* = 300)	*P*	WT (*n* = 79)	Mutation (*n* = 287)	*P*
Age at diagnosis (years)						
<55	59 (18.3)	264 (81.7)	0.117	67 (20.7)	256 (79.3)	0.283
≥55	7 (16.3)	36 (83.7)		12 (27.9)	31 (72.1)	
Sex						
Female	44 (18.6)	192 (81.4)	0.682	53 (22.5)	183 (77.5)	0.584
Male	22 (16.9)	108 (83.1)		26 (20.0)	104 (80.0)	
Family history						
No	65 (18.5)	287 (81.5)	0.480	78 (22.2)	274 (77.8)	0.318
Yes	1 (7.1)	13 (92.9)		1 (7.1)	13 (92.9)	
Histologic variants (PTC)						
Classic	60 (16.9)	295 (83.1)		71 (20.0)	284 (80.0)	
Follicular	1 (25.0)	3 (75.0)	0.007	2 (50.0)	2 (50.0)	0.000
Other variants	5 (71.4)	2 (28.6)		6 (85.7)	1 (14.3)	
Tumor size (cm)						
<2	44 (15.6)	238 (84.4)	0.018	55 (19.5)	227 (80.5)	0.139
≥2	21 (27.3)	56 (72.7)		21 (27.3)	56 (72.7)	
Multifocality						
Unifocal	41 (23.2)	136 (76.8)	0.012	43 (24.3)	134 (75.7)	0.106
Multifocal	24 (13.0)	160 (87.0)		32 (17.4)	152 (82.6)	
Lesion location						
Unilateral	40 (21.6)	145 (78.4)	0.071	49 (26.5)	136 (73.5)	0.021
Bilaterality	26 (14.4)	155 (85.6)		30 (16.6)	151 (83.4)	
Extrathyroidal extension						
No	44 (18.2)	198 (81.8)		50 (20.7)	192 (79.3)	
Minimal	14 (14.4)	83 (85.6)	0.191	18 (18.6)	79 (81.4)	0.039
Gross	8 (29.6)	19 (70.4)		11 (40.7)	16 (59.3)	
Capsular invasion						
No	30 (18.2)	135 (81.8)	0.964	33 (20.0)	132 (80.0)	0.489
Yes	36 (18.0)	164 (82.0)		46 (23.0)	154 (77.0)	
N stage						
N1a	31 (16.8)	154 (83.2)	0.521	36 (19.5)	149 (80.5)	0.318
N1b	35 (19.3)	146 (80.7)		43 (23.8)	138 (76.2)	
Number of LNM						
≤5	28 (13.5)	179 (86.5)	0.009	37 (17.9)	170 (82.1)	0.042
>5	38 (24.2)	119 (75.8)		42 (26.8)	115 (73.2)	
Size of the largest metastatic focus to LNM (cm)						
<0.65	25 (11.0)	202 (89.0)	0.000	31 (13.7)	196 (86.3)	0.000
≥0.65	40 (30.5)	91 (69.5)		46 (35.1)	85 (64.9)	
Extranodal extension						
No	38 (15.8)	203 (84.2)	0.118	48 (19.9)	193 (80.1)	0.282
Yes	28 (22.4)	97 (77.6)		31 (24.8)	94 (75.2)	
Cumulative iodine dose (MBq)						
0	7 (12.5)	49 (87.5)		14 (25.0)	42 (75.0)	
≤5550	39 (18.4)	173 (81.6)	0.460	40 (18.9)	172 (81.1)	0.332
>5550	20 (20.4)	78 (79.6)		25 (25.5)	73 (74.5)	
Distant metastasis						
No	59 (16.6)	296 (83.4)	0.000	71 (20.0)	284 (80.0)	0.000
Yes	7 (63.6)	4 (36.4)		8 (72.7)	3 (27.3)	
Response to therapy						
Excellent response	26 (13.9)	161 (86.1)	0.394	34 (18.2)	153 (81.8)	0.106
Not cured^a^	40 (22.3)	139 (77.7)		45 (25.1)	134 (74.9)	

^a^Not cured include Indeterminate, Biochemical and Structural incomplete response.

PTC, papillary thyroid cancer; LNM, lymph node metastasis; WT, wild type.

**Table 4:** Multivariate analysis of relationships between clinical features and BRAF V600E in primary tumors and LNM.

Characteristics	BRAF V600E in primary tumors		BRAF V600E in LNM	
	OR (95% CI)	*P*	OR (95% CI)	*P*
Histologic variants	0.500 (0.210–1.188)	0.117	0.319 (0.113–0.895)	0.030
Tumor size (cm)	0.594 (0.303–1.163)	0.128	0.769 (0.390–1.517)	0.449
Multifocality	2.026 (0.954–4.304)	0.066	1.166 (0.572–2.376)	0.673
Lesion location	1.159 (0.547–2.458)	0.700	1.882 (0.908–3.901)	0.089
Extrathyroidal extension	0.946 (0.489–1.830)	0.869	0.756 (0.399–1.431)	0.390
Capsular invasion	1.385 (0.664–2.889)	0.385	1.200 (0.588–2.449)	0.617
Distant metastasis	0.201 (0.041–0.993)	0.049	0.125 (0.023–0.668)	0.015
Risk stratification	1.094 (0.432–2.771)	0.850	1.017 (0.423–2.445)	0.970
Cumulative iodine dose	1.284 (0.770–2.141)	0.338	1.614 (0.982–2.653)	0.059
Response to therapy	0.859 (0.439–1.681)	0.657	1.055 (0.554–2.010)	0.870
N stage	1.797 (0.878–3.681)	0.109	1.795 (0.902–3.573)	0.096
Number of LNM	0.607 (0.292–1.260)	0.180	0.670 (0.332–1.355)	0.265
Size of the largest metastatic focus to the LN (cm)	0.297 (0.143–0.616)	0.001	0.242 (0.119–0.492)	0.000
Extranodal extension	1.100 (0.548–2.208)	0.788	1.540 (0.775–3.061)	0.218

LNM, lymph node metastasis; PTC, papillary thyroid cancer.

## Discussion

In this study, the BRAF V600E mutation was evaluated in both the primary tumor and LNM in PTC patients. The BRAF V600E mutation was slightly higher in primary tumors than in LNM (81.5% vs 78.0%), and the concordance of the genotype between primary tumors and LNM was high (ICC = 0.865). Other studies detected BRAF V600E mutations in primary tumors in 48.5–83.7% of PTC cases, and BRAF V600E mutation frequency has increased in recent years (4, 6, 17, 18). The BRAF V600E positivity in the current study is similar to the previous reports both in primary tumors (19, 20) and in LNM (21, 22). Some studies (9, 21) found high concordance of the genotype between primary tumors and LNM in BRAF V600E mutations. Their results suggested that local metastasis (such as LNM) was not indicative of new molecular changes or due to the presence of the specific molecular changes, such as BRAF V600E or RAS mutations, but more closely related to the morphological characteristics of tumors (23, 24, 25). Lin *et al.* (26) showed that the genetic characteristics of primary tumors were basically consistent with local metastases but with some differences that can be explained by the selection of mutant alleles during tumor progression or the heterogeneous pattern of tumoral cells in primary tumor with only subclones having the ability to metastasize (21).

In the current study, the BRAF V600E mutation was higher in the classical PTC subtype than in the other subtypes; however, the number of other subtypes were very small. A previous study done by our group, as well as the work of Sancisi *et al.*, showed that the BRAF V600E mutation occurred less often in the invasive PTC subtype than in the classic subtype in PTC patients without distant metastases (8, 27), and the prevalence of the BRAF V600E mutation was lower in follicular variant than in conventional PTC (28), although Straccia *et al.* reported the opposite result (29). Criteria for inclusion and exclusion differed among the studies, which may have led to different results. Some researchers suggested an association between molecular genotype and histological subtype (30).

PTC patients with higher number of LNM showed higher BRAF V600E mutation frequency in primary tumors and LNM, although it was not an independent risk factor in our study. Some previous studies also found that BRAF V600E mutation in primary tumors was not an independent indicator of LNM and that BRAF V600E mutation did not correlate with LNM (31, 32). Li *et al.* observed that BRAF V600E mutation carriers were less likely to present level V LNM than the patients with BRAF V600E WT (33). In addition, we found that the size of the largest metastatic focus of LNM negatively correlated with the occurrence of the BRAF V600E mutation in both the primary tumor and LNM. Indeed, when the size of the largest metastatic focus of LNM was ≥0.65 cm, the BRAF V600E mutation frequency was lower. In contrast, Kurtulmus *et al.* showed that BRAF V600E-positive metastatic lymph nodes were greater in diameter than those without the BRAF V600E mutation (10). Different results may be due to the remarkably lower overall BRAF V600E positivity in LNM in their study (47.1%, 24/51) compared with our study (78.0%, 291/373) and other studies (73.9, 81.0%) (21, 22). Different results may also stem from differences in the studied populations. Moreover, Kurtulmus *et al.* did not conduct multivariate analysis, which may increase the risk of the effects of confounding factors. The invasive characteristics of LNM (number, extranodal extension, and stage) and clinical outcomes were not independent risk factors for the BRAF V600E mutation in LNM in our study.

Patients with larger size, multifocal lesions, bilateral lesions, and extrathyroidal extension showed higher BRAF V600E mutation frequency in primary tumors or LNM, but they were not independent risk factors in our study. The BRAF V600E mutation has been reported to be associated with aggressive characteristics and poor prognosis in PTC (4, 5, 6); however, there are different opinions in the literature. Some studies have reported that the BRAF V600E mutation is not associated with more extensive or aggressive clinicopathological features (such as extrathyroidal invasion) and is not predictive of recurrence or survival in PTC (19, 31, 32, 34, 35, 36). In fact, the BRAF V600E mutation frequency in primary tumors and LNM was lower in PTC patients with distant metastases than in patients without distant metastases in our study, which was similar to the results of previous studies that suggested that the BRAF V600E mutation did not confer a metastatic advance to cancer cells (8, 9). Thus, the BRAF V600E mutation as a prognostic biomarker for PTC remains a matter of debate and may be dependent on the population studied (36). A study by Randolph *et al.* and a previous study conducted by our group (15, 16) both showed that the prognosis depended on the size, number and extranodal extension of LNM in PTC as the size of the largest metastatic focus was significantly associated with an incomplete response. Therefore, the BRAF V600E mutation may not correlate with the invasiveness of LNM; rather, the development and prognosis of LNM depend on its pathological characteristics and not on the BRAF V600E mutation.

There were some limitations to the present study. First, a relatively small number of PTC patients from a single center were retrospectively analyzed, thus, there may be a selection bias in enrolled patients in our study. However, the BRAF V600E mutation frequency in the primary tumors in this study (81.5%) was similar to previous reports (83.7%) (19, 20). Likewise, the BRAF V600E positivity in LNM was 78% in our study, which was similar to other studies (73.9, 81.0%) (21, 22). Therefore, the selection bias in this study was mild. Secondly, the follow-up time was not long enough to obtain recurrence or mortality risk. Although the initial therapy response was based on the 2015 ATA guidelines of known as dynamic risk stratification, a longer period of follow-up is necessary. In addition, obtaining tissue samples of distant metastases, such as in the lung and bone tissue, was difficult; thus, we did not examine the occurrence of the BRAF V600E mutation in distant metastases in the present study, which must be done in future research.

## Conclusion

The BRAF V600E mutation was higher in primary tumors than in LNM while the concordance of the genotype between them was high. PTC patients with the BRAF V600E mutation in LNM did not have a larger diameter of LNM and did not exhibit poor clinical outcomes. There was no relationship between the BRAF V600E mutation in LNM and the number, extranodal extension, and stage of LNM. Therefore, the BRAF V600E mutation in LNM may not be related to the invasive characteristics of LNM in PTC.

Our results strongly imply that the BRAF V600E mutation may not be used as a unique molecular determinant in treatment decisions; however, studies with higher quality and longer follow-up time are needed. We hope that more scientists will conduct further research to generate a critical mass of data on the correct use of the BRAF mutation in clinical practice.

## Declaration of interest

The authors declare that there is no conflict of interest that could be perceived as prejudicing the impartiality of the research reported.

## Funding

This work was supported by the Clinical Research Startup Program of Southern Medical University by High-level University Construction Funding of Guangdong Provincial Department of Education (grant number LC2019ZD025, 2019) and Guangdong Province Science and Technology Plan Projects (grant number 2019A141405043, 2019).
